# α-Lipoic acid: a potential regulator of copper metabolism in Alzheimer’s disease

**DOI:** 10.3389/fmolb.2024.1451536

**Published:** 2024-09-03

**Authors:** Sigrid Kirss, Anette Reinapu, Ekaterina Kabin, Julia Smirnova, Vello Tõugu, Peep Palumaa

**Affiliations:** Department of Chemistry and Biotechnology, Tallinn University of Technology, Tallinn, Estonia

**Keywords:** Alzheimer’s disease, copper metabolism, metalloneurochemistry, alphalipoic acid, SH-SY5Y cell line

## Abstract

Alzheimer’s disease (AD) is characterized by classic hallmarks such as amyloid plaques and neurofibrillary tangles, however, intensive research has broadened its scope to explore additional underlying mechanisms. Notably, disruptions in metal homeostasis, particularly involving copper, have gained significant attention. In AD pathology, an imbalance is evident: there is an excess of extracellular copper alongside a deficiency in intracellular copper in brain tissue. Our previous work demonstrated that α-lipoic acid (LA) can effectively shift copper from the extracellular space to the intracellular environment in a neuronal cell model. However, the precise mechanism of action and role of LA in copper metabolism remained elusive. In this study, we compared the cellular effects of LA with those of different synthetic copper-binding ligands: diethyldithiocarbamate (DETC), clioquinol (CQ), D-penicillamine (D-PA) and elesclomol (ES). Using differentiated SH-SY5Y cell culture as a neuronal model, we found that, unlike other synthetic compounds, natural ligand LA is not toxic in the presence of extracellular copper, even at high doses. LA gradually increased intracellular copper levels over 24 h. In contrast, DETC, CQ, and ES acted as fast copper ionophores, potentially explaining their higher toxicity compared to LA. D-PA did not facilitate copper uptake into cells. We demonstrated that a slow increase of LA inside the cells is enhanced in the presence of copper. Furthermore, the ability of LA to modulate the equilibrium of extra- and intracellular copper was evident when we added copper isotope ^65^Cu. The ratio of copper isotopes changed rapidly, reflecting the impact of LA on the equilibrium of copper distribution without affecting the copper transport network. Our results provide compelling evidence that α-lipoic acid holds promise as a non-toxic agent capable of normalizing copper metabolism in Alzheimer’s disease.

## Introduction

Alzheimer’s disease (AD) is a neurodegenerative disorder characterized by amyloid-beta plaques and neurofibrillary tangles in the brain first described by Alois Alzheimer (Alzheimer, 1906). Currently, AD stands as the most common cause of dementia, affecting over 55 million people worldwide (International, 2019). AD is becoming one of the most burdensome, costly, and lethal diseases of this century (Europe, 2021) as its prevalence is predicted to double in Europe and triple globally by 2050 (Scheltens et al., 2021). Despite these alarming statistics, there is still no definitive treatment for AD, although substantial efforts have been directed toward developing better treatment and prevention strategies.

While amyloid plaques and neurofibrillary tangles continue to be the defining features of AD (Hardy and Higgins, 1992; Selkoe and Hardy, 2016), research has expanded its focus to explore other underlying mechanisms. Inflammation and disruptions in metal homeostasis are now under scrutiny (Bagheri et al., 2017). Most therapeutic approaches aim to modify the disease by targeting amyloid-beta (Aβ) peptide and tau protein, or by addressing cognitive symptoms (Long and Holtzman, 2019). Notably, the FDA has approved two promising anti-amyloid monoclonal antibodies Aducanumab and Lecanemab (Mullard, 2021; Verger et al., 2023), although conflicting evidence exists regarding the therapeutic benefits of these expensive treatments (Brockmann et al., 2023). Consequently, the search for alternative therapies and more effective methods to prevent AD progression continues.

Mounting evidence highlights a close association between AD and copper metabolism imbalance within the brain (Bulcke et al., 2017; Chiorcea-Paquim et al., 2018). Copper, an essential trace element, serves as a vital redox cofactor for over twenty enzymes. Its multifaceted roles include participation in oxidative metabolism (via various oxidases), cellular respiration (through cytochrome c oxidase, CcO), and antioxidant defence (via Cu, Zn-superoxide dismutase, Cu,Zn-SOD1). However, excess “free” copper ions can have detrimental effects, generating reactive oxygen species (ROS) (Sies et al., 2017) or triggering regulated cell death through a mechanism known as cuproptosis (Tsvetkov et al., 2022). Thus, maintaining precise cellular copper levels is critical, and it is facilitated by a sophisticated protein network that strictly controls copper transport and reactivity. Dysregulation of copper metabolism leads to various diseases, exemplified by Menkes disease (MD) and Wilson’s disease (WD) which are caused by loss-of-function mutations in the copper transporter ATP7A or ATP7B, respectively. At the organismal level, there is a systemic copper deficiency in the case of MD, and accumulation in the case of WD, which can be treated with copper supplements or chelators, respectively (Bull et al., 1993).

In AD, significant elevation of copper levels occurs in extracellular spaces (Hozumi et al., 2011; Li et al., 2017; Sensi et al., 2018; Squitti et al., 2021; Squitti et al., 2021) including blood serum and cerebrospinal fluid (CSF), alongside with concurrent intracellular copper deficiency in brain tissue (Schrag et al., 2011; Squitti et al., 2021; Xu et al., 2017). The “CuAD hypothesis” suggests that this copper imbalance, resulting from aging and AD pathology, contributes to neurodegeneration by causing extracellular oxidative stress and intracellular misfunctioning of copper enzymes such as CcO and Cu,Zn-SOD1 (Squitti et al., 2021). Thus, normalization of copper homeostasis in the brain may provide a therapeutic outcome. The best approach involves redistributing copper from the extracellular environment to the intracellular milieu (Lei et al., 2021). This task can be accomplished by copper ionophores which facilitate transmembrane transport of copper ions, but also by other copper-binding ligands affecting the balance of copper distribution. Our recent study demonstrated that the natural compound alpha-lipoic acid (LA), also known as thioctic acid, enhances copper transport into cultured neuron-like cells (Metsla et al., 2022). However, the precise mechanism underlying LA action remained elusive.

LA serves as a naturally occurring cofactor in various multienzyme complexes in both prokaryotic and eukaryotic cells (Bast and Haenen, 2003). These complexes include pyruvate dehydrogenase, α-ketoglutarate dehydrogenase, branch-chained α-ketoacid dehydrogenase, and 2-oxoadipate dehydrogenase (Theodosis-Nobelos et al., 2021). In the human organism, LA is synthesized in small quantities within the mitochondrion from octanoic acid, however, its primary source is through the exogenous intake (Salehi et al., 2019). Beyond its role as an antioxidant, LA also exhibits metal-binding properties (Bjorklund et al., 2019; Ou et al., 1995; Goralska et al., 2003; Jalili-Baleh et al., 2021). Our research group previously demonstrated that a reduced form of LA, dihydro-LA (DHLA), effectively binds Cu (I) ions. Its Cu (I)-binding affinity lies between that of glutathione and intracellular copper chaperones and enzymes (Smirnova et al., 2018; Banci et al., 2010). Notably, LA can cross the blood-brain barrier (BBB) (Bilska et al., 2007), making it a promising candidate for modulating copper metabolism.

In our study, we aimed to understand the mechanism behind LA’s impact on copper distribution. We compared the cellular effects of LA with those of different synthetic copper-binding ligands, such as clioquinol (CQ), elesclomol (ES), D-penicillamine (D-PA), and diethyldithiocarbamate (DETC). CQ is a copper ionophore, which has been previously tested in AD clinical trials (White et al., 2006; Treiber et al., 2004) but had toxic side effects (Kawamura et al., 2014). ES is also a copper ionophore, which induces cuproptosis (Tsvetkov et al., 2022). D-PA is a copper chelator widely used in WD therapy (Brewer et al., 1987; Brewer 1999; Chen et al., 2012). DETC is a copper ionophore which leads to a substantial increase in intracellular copper (Cheng and Trombetta 2004). Using differentiated SH-SY5Y cell culture as a neuronal model, we evaluated the toxicity of these compounds in the presence of elevated extracellular copper. Remarkably, LA proved non-toxic even in the presence of copper at high doses, distinguishing it from the synthetic counterparts. Additionally, we observed LA incorporation into cells and its impact on the equilibrium between extracellular and intracellular copper, as well as assessed its impact on the cell proteome.

## Materials and methods

### Cultivation of cells

Human SH-SY5Y neuroblastoma cells (ATCC) were grown in Dulbecco’s Modified Eagle Medium without Phenol Red (DMEM) (21063–029, Gibco) supplemented with 10% fetal bovine serum (FBS; A5256701, Gibco) and 1% penicillin and streptomycin (PEST; 15140, Gibco) at 37°C in a humidified atmosphere containing 5% CO_2_. The culture medium was changed every 2–3 days and the cells were split every 5–7 days using 0.25% Trypsin-EDTA (25200, Gibco), where the passage number did not exceed p20.

SH-SY5Y cells were differentiated in cell culture plates, using the differentiation protocol described in (Krishtal et al., 2019). Briefly, cells were pre-differentiated with 10 μM retinoic acid (RA; R2625, Sigma-Aldrich) in complete medium for 4 days (day 1–4), followed by differentiation with 50 ng/mL brain-derived neurotrophic factor (BDNF; B-250, Alomone Labs) in serum-free medium for 2 days (day 5–7).

### Toxicity experiments

For toxicity experiments, cells were grown and differentiated at the initial density of 1.8 × 10^5^ cells/mL in white, clear bottom 96-well plates. Differentiated SH-SY5Y cells were treated for 24 h with different concentrations of freshly prepared D-PA (P4875, Sigma-Aldrich), DETC (318116, Sigma-Aldrich), CQ (24880, Sigma-Aldrich), ES (SML2651, Sigma-Aldrich) or LA (T5625, Sigma-Aldrich), alone or in the presence of 5 μM CuCl_2_ (Sigma-Aldrich). For the assessment of cell viability with propidium iodide (PI) (81845, Fluka), 0.5 mM PI in phosphate-buffered saline (PBS) (P4417, Sigma) was added to each well to the final concentration of 2.5 μM, and incubated for 10 min at 37°C. TECAN Genios Pro microplate reader was used to measure fluorescence (excitation 540 nm, emission 612 nm), as described previously (Krishtal et al., 2019).

### Inductively coupled plasma mass spectrometry (ICP-MS) analysis

Ultrapure Milli-Q-quality water with a resistivity of 18.2 MΩ/cm, produced by a Merck Millipore Direct-Q and Direct–Q UV water purification system (Merck KGaA, Darmstadt, Germany), was used for all sample preparations.

For ICP-MS experiments, cells were plated at a density of 2 × 10^5^ cells/mL in opaque, clear-bottom 6-well plates (Greiner Bio-One), and differentiated as described above. For data normalisation, cells from each sample were counted in the Countess Automated Cell Counter (Invitrogen) with Countess™ Cell Counting Chamber Slides using trypan blue (C10228, Invitrogen) method. For ^63^Cu content measurements using ICP-MS, differentiated SH-SY5Y cells were treated for 1 h, 5 h and 24 h with 5 μM CuCl_2_ in the presence of 20 μM LA, 1 μM and 100 nM DETC, 100 nM CQ, 50 μM D-PA, and 10 nM ES. To measure copper isotopes, cells were treated with 5 μM CuCl_2_ in the presence of 20 μM LA, after 12 h the medium was changed to containing 5 μM ^65^Cu in the presence of 20 μM LA. ^65^CuO (>99% ^65^Cu, Cambridge Isotope Laboratories) was dissolved by stirring in concentrated HCl (258148 Fluka) and diluted in Milli-Q-quality water to prepare a 10 mM stock solution of ^65^CuCl_2_.

Differentiated SH-SY5Y cells were treated with different concentrations of LA, DETC, CQ, ES and D-PA as described above and were collected from 6-well plate wells into acid-washed 2 mL Eppendorf tubes. Cells were pelleted and washed twice with PBS, after the supernatant was discarded. The obtained cell pellets were stored at −80°C until cell digestion.

Samples were digested by adding 100 μL of 68% HNO_3_ (A509 Trace Metal™, Fisher Chemical) to the cell pellets and incubated for 24 h at room temperature (RT). For ICP-MS analysis, samples were diluted to 3.4% HNO_3_.

Quantification of ^63^Cu and ^65^Cu was performed on an Agilent 7,800 series ICP-MS instrument (Agilent Technologies, Santa Clara, United States), and an Agilent SPS-4 autosampler was used for sample introduction. For instrument control and data acquisition, ICP-MS MassHunter 4.4 software Version C.01.04 from Agilent was used. ICP-MS analysis was performed in peak-hopping mode, six points per peak, 100 scans per replicate, 3 replicates per sample, and the instrument was operated under general matrix working mode under the following conditions: RF power 1,550 W, nebulizer gas flow 1.05 L/min, the plasma gas flow 15 L/min, nebulizer type: MicroMist. The measurements were performed in He mode. The ICP-MS apparatus was calibrated using multielement calibration standard solutions in the range of 0.50–50 ppb in 3.4% trace metal grade HNO_3_ (8,500–6,940, Agilent Technologies, United States) containing Ag, Al, As, Ba, Be, Ca, Cd, Co, Cr, Cs, Cu, Fe, Ga, K, Li, Mg, Mn, Na, Ni, Pb, Rb, Se, Sr, Tl, U, V, Zn. The internal standard for ^63^Cu and ^65^Cu was ^72^Ge (5,188–6,525, ICP-MS internal standard mix 1 μg/mL in 2% HNO_3_, Agilent Technologies).

### High-performance liquid chromatography (HPLC) analysis

For HPLC analysis, cells were grown and differentiated as described for ICP-MS analysis. To measure LA content with HPLC, differentiated SH-SY5Y cells were treated for 2 h, 5 h and 24 h with 5 μM CuCl_2_ in the presence of 20 μM and 50 μM LA. Cells were collected into 2 mL Eppendorf tubes and centrifuged for 1 min at 10,000 rpm to pellet the cells, and the supernatant was discarded. For data normalization, cells were counted as described above. Counted cells were washed twice with PBS, mixed using a vortex mixer, and centrifuged for 3 min at 10,000 rpm. Next, 60 μL of Milli-Q-quality water was added to Eppendorf tubes with cells, mixed using a vortex mixer, and the cell suspension was then pipetted into 0.1 mL PCR tubes. Cell samples were stored at −80°C until the HPLC analysis. Before the analysis, cells were sonicated with BioRuptor Pico (Diagenode) (30 s on/off, 15 cycles).

The protocol for sample preparation was adapted from Satoh et al. (2007). Briefly, 10 μL of 160 mM tris (2-carboxyethyl)phosphine (TCEP) (A0410501, Acros Organics) was added to the cell lysates to reduce disulfide bonds in proteins, and incubated for 10 min. Samples were acidified with 1 μL of TFA (2191578, Riedel-de Haën) and incubated for 5 min, followed by 60 μL of acetone:ethyl acetate 1:1 mixture (Acetone EtOAc). Samples were centrifuged for 5 min at 15,000 rpm, and the upper layers of the supernatant were collected and transferred to clean Eppendorf tubes. This step was repeated 3 times, after which the samples were placed in a vacuum desiccator for 24 h. 60 μL of 0.1 M sodium tetraborate (STB) (206291000, Acros Organics) containing 2 mM EDTA (pH = 9.4) was added to the completely dried samples. Further, thiol groups were reduced with 10 μL of 160 mM TCEP, and labelled with 10 μL of 40 mM 4-(aminosulfonyl)-7-fluoro-2,1,3-benzoxadiazole (ABD-F; FA17851, Biosynth). The samples were incubated for 10 min at RT and centrifuged for 10 min at 15,000 rpm. 60 μL of the analyte was injected into HPLC cone vials (Macherey-Nagel) and inserted into the instrument. ABD-F calibration curve, covering a range of 0.5–6 μM LA was created before every experiment (Supplementary Figure 1).

A Shimadzu HPLC system consisting of two LC-30AD pumps, a CBM-20A system controller, an autosampler (SIL-30AC), and a degasser (DGU-20A5R) was used. The analytical column for reversed-phase chromatography was Kinetex 2.6 μm C18 100Å (150 mm × 4.6 mm i.d.; Phenomenex). The column was maintained at 40°C by a CTO-20AC column oven (Shimadzu) and the peaks were detected with SPD-20A UV/VIS and RF-20AXS fluorescence detectors. The fluorescence detector was set at 380 nm (excitation) and 510 nm (emission). A linear gradient elution from H_2_O-CH_3_CN (95:5) containing 0.1% TFA to H_2_O-CH_3_CN (5:95) containing 0.1% TFA 0%–50% over 30 min was applied. The flow rate of the mobile phase was 0.5 mL/min. The peak areas obtained from both detectors were calculated using LabSolutions software version 5.42 (Shimadzu).

### LC-MS/MS analysis, peptide identification and quantification

For proteomics experiments, cells were plated at a density of 4 × 10^5^ cells/mL into a 6-cm cell dish (Greiner Bio-One), and differentiated as described above. Differentiated SH-SY5Y cells were treated with 20 μM LA and 5 μM CuCl_2_ for 24 h, alone or in combination.

Cells were collected into clean 15 mL tubes, pelleted, and washed twice with PBS. The supernatant was discarded. The obtained cell pellets were stored at −80°C until cell digestion.

Samples were lysed with 4% SDS, 10 mM Tris-HCl pH 7.5, 50 mM DTT, heated for 5 min at 95°C, sonicated with 3 × 20 s pulses, and pelleted by centrifugation at 17,000 g for 10 min at 4°C. 10 μL of supernatant was transferred to a fresh Protein LoBind sample tube and precipitated with TCA-DOC. The concentration of the precipitated protein was measured using the Micro BCS assay (Thermo Fisher Scientific). 15 μg of the resulted protein pellet was suspended with 7 M urea, 2 M thiourea, 100 mM ABC, 20 mM methylamine buffer to the final concentration of 0.5 μg/μL 100 mM DTT was added to the protein solution, vortexed, and incubated at RT for 1 h. Next, CAA was added to the final concentration of 10 mM, vortexed, and incubated in the dark for 1 h. Samples were further digested with proteomics grade Lys-C (Wako) for 1 h, further diluted with 100 mM ABC and supplemented with dimethylated porcine trypsin (Sigma Aldrich) for 18 h digestion. Next, the peptides were acidified with TFA to the final concentration of 1.0%, vortexed, and purified with 3x-layer C18 StageTip (3M Empore). As the last step, the peptides were suspended with sonication with 0.5% TFA, 0.05 ng/μL of iRT solution to the final concentration of 500 ng/μL. Peptides (1 µg) were separated in a 50 cm × 75 μm ID emitter column (New Objective, United States), packed with 3 μm ReproSil-Pur C18AQ (Dr. Maisch, Germany) and attached to Thermo Fisher Scientific Easy-nLC 1,000. A linear 120 min 5%–35% gradient of solvent B (flow rate 250 nL/min) was used to elute the peptides (Buffer A: 0.1% formic acid, buffer B: 80% acetonitrile +0.1% formic acid). The peptides were detected with a Thermo Fisher Scientific Q Exactive Plus mass spectrometer. Each 350–1,400 m/z MS scan at a resolution setting 70,0000 was followed by MS/MS analysis of up to 10 most intense peaks.

Raw data files were processed using MaxQuant software package (version 1.6.15.0) and default settings, and a peptide identification search was performed against the human reference proteome database downloaded on 20.09.2020 from the UniProtKB database.

### Bioinformatic analysis

Ratios of the protein abundance mean values and p-values were used for generating volcano plots in R-studio (ggplot2 package). Proteins with more than two-fold change (log_2_ > 1) and *p*-value <0.05 were considered as significantly changed, proteins with more than 0.5-fold change (log_2_ > 0.05 < 1) and *p*-value <0.05 were considered as potentially affected by the treatment.

### Statistical analyses

GraphPad Prism 10 was used for data visualization and statistical analysis. Data from PI assay and ICP-MS were analysed using a one-way analysis of variance (ANOVA) with the *post hoc* Dunnett’s multiple comparison test. All cell experiments were done in 3 replicates and repeated 3–4 times. Data on graphs are presented as mean ± standard error of the mean (SEM). A p-value of less than 0.05 was considered statistically significant.

## Results

### LA is not toxic to the differentiated SH-SY5Y cells in the presence of copper in contrast to DETC, CQ, D-PA and ES

The human neuroblastoma cell line SH-SY5Y serves as a valuable cellular model widely used in biochemical and toxicological studies of neurodegenerative diseases. Upon differentiation with RA and BDNF, these cells extend long neurites and create neuronal networks, resulting in a cell culture that resembles neurons (Krishtal et al., 2019). To assess copper toxicity, differentiated SH-SY5Y cells were incubated with different concentrations of LA, DETC, CQ, D-PA or ES in the presence of CuCl_2_ for 24 h.

**FIGURE 1:**
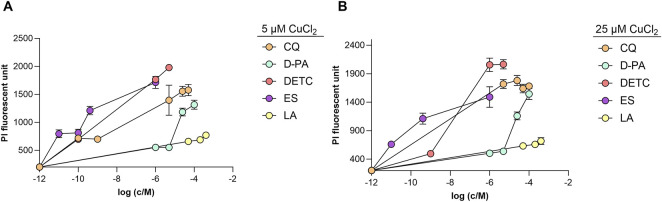
Toxicity of the selected copper-binding ligands to differentiated SH-SY5Y cells in the presence of copper (II) ions. Cells were incubated for 24 h with different concentrations (1 nM–400 μM) of CQ, D-PA DETC, ES and LA in the presence of 5 μM CuCl_2_ **(A)** and 25 μM CuCl_2_ **(B)**. PI fluorescence was measured at 612 nm (excitation 540 nm). Data is shown mean ± SEM; n = 3–12.

Initially, we demonstrated that DETC, CQ, D-PA, ES, and Cu (II) ions at their tested concentration ranges did not exhibit individual toxicity in the cell culture (Supplementary Figures 2, 3). In our recent study, we demonstrated that supplementation of LA individually was not toxic to the differentiated SH-SY5Y cell culture (Metsla et al., 2022). To assess the potential toxicity of copper chelators in the presence of exogenous copper, we selected two moderate concentrations of CuCl_2_: 5 μM, and 25 μM. All synthetic copper-binding compounds exhibited cellular toxicity when combined with copper supplementation. ES showed the highest toxicity starting with nanomolar concentrations, DETC was toxic starting from micromolar concentration, CQ was found to be toxic at submicromolar concentrations. D-PA exhibited moderate toxicity after reaching 200 μM in the presence of 5 μM copper. In contrast, LA was not toxic at up to 600 μM (Figure 1). However, the combination of very high concentrations of copper and LA (400 μM LA and 50 μM CuCl_2_) had a toxic effect (Supplementary Figure 4).

### LA can redistribute exogenous copper into a cell in a safe manner

Previously, we reported that LA can shift copper from the extracellular to the intracellular environment (Metsla et al., 2022). To establish the kinetics of this phenomenon, we treated differentiated SH-SY5Y cells with LA in the presence of copper for 1 h, 5 h, and 24 h and compared its effect with those of DETC, CQ, D-PA, and ES using ICP-MS.

**FIGURE 2:**
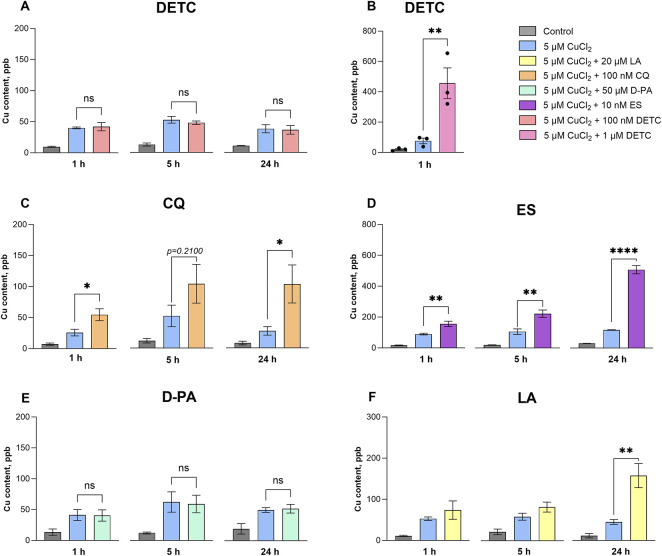
Influx of copper into differentiated SH-SY5Y cells after treatment with the selected copper-binding ligands. Differentiated SH-SY5Y cells were treated with 100 nM DETC **(A)**; 1 μM DETC **(B)**; 100 nM CQ **(C)**; 10 nm ES **(D)**; 50 μM D-PA **(E)** and 20 μM LA **(F)** in the presence of 5 CuCl_2_ for 1 h, 5 h and 24 h. Data are shown as mean ± SEM; n = 3–4. One-way ANOVA followed by a Dunnett’s multiple comparisons test at the 0.05 level was used for statistical analysis. Main effect of treatment ∗∗∗∗ *p* < 0.0001; ∗ *p* < 0.05; n. s., not significant with respect to the cells treated with 5 μM CuCl_2_.

We found that a sub-toxic concentration of DETC, 100 nM, did not induce copper influx (Figure 2A). In contrast, supplementation of the cells with 1 μM DETC for 1 h caused a significant increase in intracellular copper content (Figure 2B), and after 5 h of incubation all cells died. This result suggests that micromolar DETC transports excessively high concentrations of exogenous copper into the cells, leading to copper-induced cell death. CQ and ES similarly cause a rapid copper uptake (Figures 2C, D), resulting in a drastic increase in cellular copper content, which leads to their toxic trait. DETC, CQ, and ES are recognized as copper ionophores, and our data strongly support their mechanism of action.

In contrast, D-PA, which is used as a copper chelator in WD therapy, did not cause an elevation of intracellular copper (Figure 2E), aligning with its chelating nature. Compared to other compounds, LA caused a slower and more moderate increase of cellular copper, with the major effect observed after 24 h (Figure 2F).

### Differentiated SH-SY5Y cells uptake LA in the presence of copper

**FIGURE 3:**
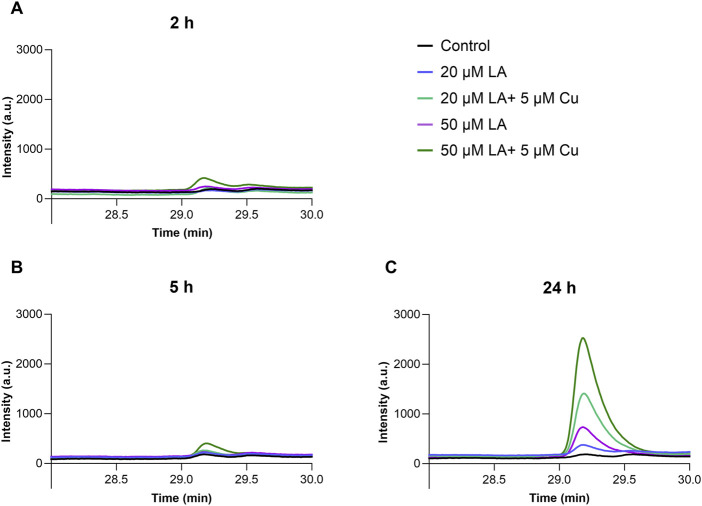
Elevation of LA concentration in differentiated SH-SY5Y cells in the presence of copper ions. SH-SY5Y cells were treated for 2 h **(A)**, 5 h **(B)** and 24 h **(C)** with 50 μM LA (purple) and in presence of 5 μM CuCl_2_ (dark green); with 20 μM LA (blue) and in presence of 5 μM CuCl_2_ (light green); control cells (black). Representative ABD-F-LA chromatogram measured in SH-SY5Y cells. Chromatographic conditions: Kinetex 2.6u C18 100A column, 30 min linear gradient 0%–50% B using H_2_O-CH_3_CN (5:95) and H_2_O-CH_3_CN (95:5) solutions containing 0.1% trifluoroacetic acid (TFA). Flow rate 0.5 mL/min. Fluorescence intensity was measured with excitation at 380 nm and emission at 510 nm.

Copper ionophores are known to increase cellular copper content by entering the cell as copper-bound complexes (Ding and Lind, 2009). To clarify, whether LA acts through a similar mechanism, we used HPLC analysis to measure LA concentration in the cells after 2 h, 5 h, and 24 h of treatment. We found that LA was detectable in the cells after 2 h and 5 h of incubation (Figures 3A, B), and its content increased after 24 h incubation in a concentration-dependent manner. In the presence of supplemented copper, cellular concentrations of LA increased (Figure 3C), reaching 34 μM level after 24 h treatment with 50 μM LA and 5 μM CuCl_2_. Elevation of cellular copper and LA content after simultaneous treatment with these reagents provides strong evidence that LA acts as the copper ionophore.

### LA cannot reverse copper-induced effects on the cellular proteome in short-time treatment

To find out the effect of LA on the copper uptake and cellular transport machinery, we performed proteomics studies by LC-MS/MS. No changes in expression of the major copper influx protein CTR1 in cells treated with 5 μM CuCl_2_ and 20 μM LA compared to the non-treated control were detected (Supplementary Table 1). Based on the absence of changes in CTR1 expression, we could conclude that LA does not simply stimulate copper uptake by the classic transport via CTR1, but acts through distinct mechanisms. Additionally, no changes were detected in the expression of other copper-handling proteins, including copper chaperones and ATP7A, except for ATOX1 which was surprisingly significantly downregulated in Cu-treated cells and tended towards decreased expression in LA-treated cells (Supplementary Table 1).

Excess copper has been reported to affect various signalling pathways and cause disturbances in cellular functions in many studies. In our research, we aimed to reveal the effect of copper alone or in combination with LA on the neuronal cell model. As expected, not many proteins were significantly up- or downregulated after short-term treatment with adequate concentrations of Cu and/or LA (Supplementary Figure 5). Nevertheless, we found that administration of 5 μM CuCl_2_ for 24 h caused the most significant downregulation of proteins (more than 2-fold decrease) involved in cellular respiration (COX6A1), maintenance of redox balance (TMX4 and TXN2), as well as cellular morphology (TUBB8), revealing negative effects of exogenous Cu on the normal cell function even in safe doses and short period of time (Supplementary Figure 5A; Supplementary Table 2). LA supplementation, combined with Cu, was not able to rescue changes in the expression of any of these proteins (Supplementary Figures 5B, C; Supplementary Table 2). LA was previously reported to improve redox balance and upregulated synthesis of selenoproteins in cells suffering from excess copper accumulation (Kabin et al., 2023). In our dataset, we observed neither any changes in selenoproteins content nor improvements in the mitochondrial thioredoxin system (Supplementary Figures 5B–D; Supplementary Table 2; Supplementary Data Sheet 1). There could be two potential explanations for the observed differences: (i) the treatment time in our experiment was significantly shorter than in (Kabin et al., 2023), and (ii) different cell models can respond to the treatments in different ways.

Amongst the proteins influenced by copper were those involved in cell motility (MEMO1), signalling (MAPK7) and even lipid and xenobiotic metabolism (BRAP). Several candidates identified in our experiment, either in CuCl_2_-or CuCl_2_ and LA-treated groups, are directly involved in cancerogenesis (such as MEMO1, ATOX1, L1CAM, RABGGTA, MAP2K7 and others, (Supplementary Tables 1, 2) and may be of potential interest for anti-cancer research.

### LA accelerates copper intake in differentiated SH-SY5Y cells

**FIGURE 4:**
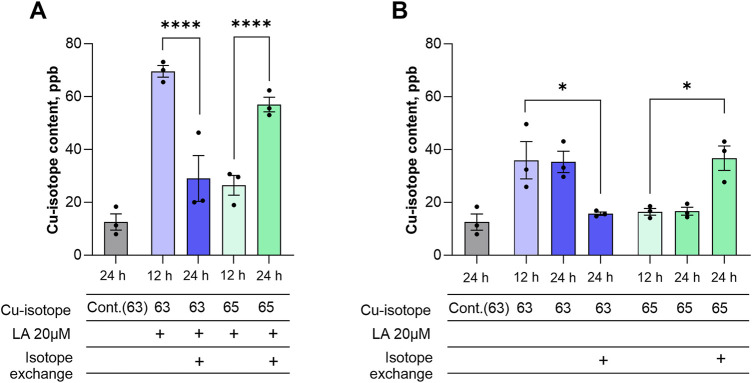
LA accelerates copper influx into differentiated SH-SY5Y cells. Differentiated SH-SY5Y cells were treated with 20 μM LA in presence of 5 μM CuCl_2_ with natural isotopic composition and after 12 h medium were changed containing 20 μM LA in presence of 5 μM Cu^65^ **(A)** and without LA **(B)**. Data are shown as mean ± SEM; n = 3. One-way ANOVA followed by a Sidak’s multiple comparisons test at the 0.05 level was used for statistical analysis. Main effect of treatment ∗∗∗∗ *p* < 0.0001.

Cellular copper accumulation can occur through two potential mechanisms: increased influx and storage of copper ions, or decreased excretion. To understand the mechanism behind the increase of the intracellular copper pool caused by LA, we performed an isotope exchange experiment monitored with ICP-MS. Cells were incubated with 5 μM CuCl_2_ with natural isotopic composition in the absence or presence of 20 μM LA. After 12 h of incubation, the cell’s medium was changed to 5 μM ^65^CuCl_2_ with or without 20 μM LA. As expected, cellular ^63^Cu concentration increased after 12 h in the presence of LA (Figure 4A). When the medium was changed to ^65^CuCl_2_ in the presence of LA, the levels of ^63^Cu dropped significantly in the following 12 h, while the levels of the ^65^Cu isotope increased (Figure 4A). Supplementing the medium with ^63^CuCl_2_ and ^65^CuCl_2_ alone resulted in the same effect with the modest Cu uptake rate compared to the copper-LA combination. Briefly, we demonstrated an increase of ^65^Cu after ^63^Cu-containing medium was changed to ^65^Cu-supplemented medium (Figure 4B). Based on the fact that isotope exchange occurs in the cells, we can conclude that copper which enters the cell mostly implements into the exchangeable copper pool rather than remaining inbound, and LA is able to increase the dynamics of this turnover.

## Discussion

Numerous hypotheses have been proposed to explain neurodegeneration occurring in AD, yet the progression curve and causative relationships between different events of the pathology have remained elusive. Copper metabolism is seriously disturbed in AD, which can contribute to the disease progression. Copper concentration is elevated in the extracellular environment, whereas cells experience copper deficiency, particularly in brain regions most affected by the disease, such as the hippocampus, amygdala, and neocortex. This copper imbalance might contribute to neurodegeneration (Squitti, Faller, et al., 2021). To address this, a copper-regulating ligand is needed to restore normal copper homeostasis in the brain. We have previously demonstrated that the natural ligand LA has a relatively strong binding affinity for Cu (I) ions (Smirnova et al., 2018), and it can redistribute copper between extracellular and intracellular environments (Metsla et al., 2022).

LA has been employed as a treatment for diabetic peripheral neuropathy in Germany for over three decades (Mijnhout et al., 2012). Beyond its established use, LA has been suggested as a potential therapeutic agent for the treatment of AD and other neurodegenerative diseases due to its neuroprotective and antioxidant properties. A recent study showed that LA improved memory impairment in rat models of AD-type dementia (Staykov et al., 2022). Furthermore, supplementation with LA has been assessed in aged Tg2576 mice overexpressing APP, demonstrating improvement in learning and memory, despite not affecting the amyloid plaque deposition (Quinn et al., 2007). However, another study showed that LA can reduce Aβ accumulation in cerebral cortex in insulin-resistant Wistar rats who were on high-fat diet (Maciejczyk et al., 2022). LA has also been found to extend the lifespan of *Drosophila melanogaster* (Bauer, Goupil, Garber, and Helfand, 2004) and immunosuppressed mice (Freisleben, Neeb, Lehr, and Ackermann, 1997). LA has been used in AD clinical trials for its antioxidant and anti-inflammatory properties: a daily dosage of 600 mg had positive effects on cognitive impairment in patients with mild AD (Hager et al., 2007; Maczurek et al., 2008). Despite these encouraging findings, further clinical trials involving LA have not been pursued. Additionally, its potential role in regulating copper metabolism remains a largely unexplored but fascinating area for further investigation.

In the current study, we further investigated the effects of LA on differentiated SH-SY5Y neuron-like cell culture and compared these with the effects of known synthetic copper-binding ligands. In contrast to other studied copper chelators, LA is not toxic to the differentiated SH-SY5Y neuron-like cell culture in the presence of elevated exogenous copper (Figure 1). LA redistributes copper to the cells rather slowly compared to the other ligands, reaching its maximum effect after 24 h (Figure 2F). We found that D-PA was cytotoxic in the presence of 5 μM Cu (II) (Figure 1), although it did not increase cellular copper content (Figure 2E). Notably, D-PA is used as a chelator for WD treatment, and it has been described that it exacerbates neurological symptoms during the early treatment phase (Litwin et al., 2015). This may be due to the neurotoxic effects of D-PA in the presence of copper, as demonstrated in our study, although it has been proposed that D-PA cannot cross the BBB (Cui et al., 2005). ES is studied mainly as an anticancer drug acting as a copper ionophore and promoting cuproptosis (Tsvetkov et al., 2022). Our results agree with the very strong and fast-acting ionophore effect of ES, manifested at nanomolar concentrations (Figure 2D), which was accompanied by toxicity in the presence of copper (Figure 1). Therefore, it is obvious that ES is not a safe candidate for normalization of copper metabolism in case of AD. CQ also caused a fast increase in cellular copper levels (Figure 2C) and was toxic to the cells. Previously, it was also reported that CQ significantly increases the intracellular level of exogenously added copper at concentrations over 20 μM within 24 h and observed toxic effect advocated against the clinical use of CQ in potential AD therapy (Katsuyama et al., 2021). We also tested DETC, which had a toxic effect on the neuron-like cells (Figure 1). Interestingly, this compound did not increase copper content below toxic level. However, at the toxic 1 μM concentration, it increased copper levels in the cells after just 1 h of treatment and caused the death of all cells within 5 h (Figures 2A, B). This indicates that DETC rapidly increases the intracellular copper levels above the critical threshold.

Analysis of the proteomes of Cu and/or LA treated cells revealed that copper supplementation had the most rapid effect on mitochondrial function and cellular redox balance, and co-administered LA was unable to rescue these changes (Supplementary Figure 5; Supplementary Table 2). We neither observed the ability of LA to induce expression of selenoproteins, as previously reported in (Kabin et al., 2023). We hypothesize that this could be due to the short treatment time or the different characteristics of the SH-SY5Y cell model compared to the reported 3T3-L1.

It is known that LA can enter the cell via sodium-dependent multivitamin transporter (SDMV) (de Carvalho and Quick, 2011). We did not see changes in the expression of SDMV in response to the treatment of cells with LA alone or combined with copper (Supplementary Table 2; Supplementary Datasheet 1), whereas the activity of SDMV should be tested in the future studies. Given that, we hypothesize that (i) LA passes the cell membrane, either passively thanks to its amphiphilic nature or via the transporter, and (ii) is reduced to DHLA intracellularly, where it binds copper and, therefore, shifts the equilibrium of copper distribution toward the intracellular space. To better understand how LA modulates copper metabolism in the cell culture, we measured LA content within cells using HPLC. Results demonstrated that LA was detected in cells after 2 h and 5 h of incubation, and the LA concentration increased further after 24 h (Figure 3). Interestingly, the cellular concentration of LA was higher in the presence of copper, suggesting that LA acts similarly to copper ionophores, as intracellular levels of LA and Cu increase reciprocally.

Proteome analysis showed no changes in the expression levels of copper transporters (such as CTR1 or ATP7A) following LA treatment (Supplementary Table 1). This indicates that their levels are not the cause of increased copper intake in the presence of LA. Copper concentration in cells can be affected a) by shifting of equilibrium between inside and outside located copper ions in conditions of fast exchange or, b) by slow increase of copper influx in conditions of slow exchange. In the experiment with copper isotopes we wanted to discriminate between these two possibilities and understand how LA increases copper concentration in the cells. We found that LA shifts fast equilibrium between inside and outside located copper ions as disproportionally more ^65^Cu isotopes were incorporated into the cells as compared with increase of intracellular copper content (Figure 4A). A previous study with 3T3-L1 preadipocytes revealed that LA causes translocation of ATP7A transporter to the secretory pathway (Kabin et al., 2023). Combining these two observations, we propose that localization of copper transporters might also play a role in the increased copper turnover influenced by LA. Therefore, further studies of the copper-handling network activity and subcellular localization of its members are required to better understand the mechanism of this phenomenon.

In summary, we demonstrated that LA is a slow-acting ligand facilitating safe copper uptake. Given its copper binding affinity without decoppering important enzymes, and its ability to cross BBB, LA could be a suitable compound for normalising copper levels in the brain. Further studies with cell lines could provide more insights on the mechanistic side of LA potential, whilst for understanding of its therapeutic effects on the organismal level animal models would be beneficial.

## Data Availability

The datasets presented in this study can be found in online repositories. The names of the repository/repositories and accession number(s) can be found in the article/Supplementary Material.
