# Supplementary material for: α-Lipoic acid: a potential regulator of copper metabolism in Alzheimer’s disease
Source: Front Mol Biosci. 2024 Sep 3;11:1451536. doi: 10.3389/fmolb.2024.1451536 (PMC11405343; doi:10.3389/fmolb.2024.1451536)
Supplement: Supplementary file 1 [file Table1.docx]

Supplementary Material


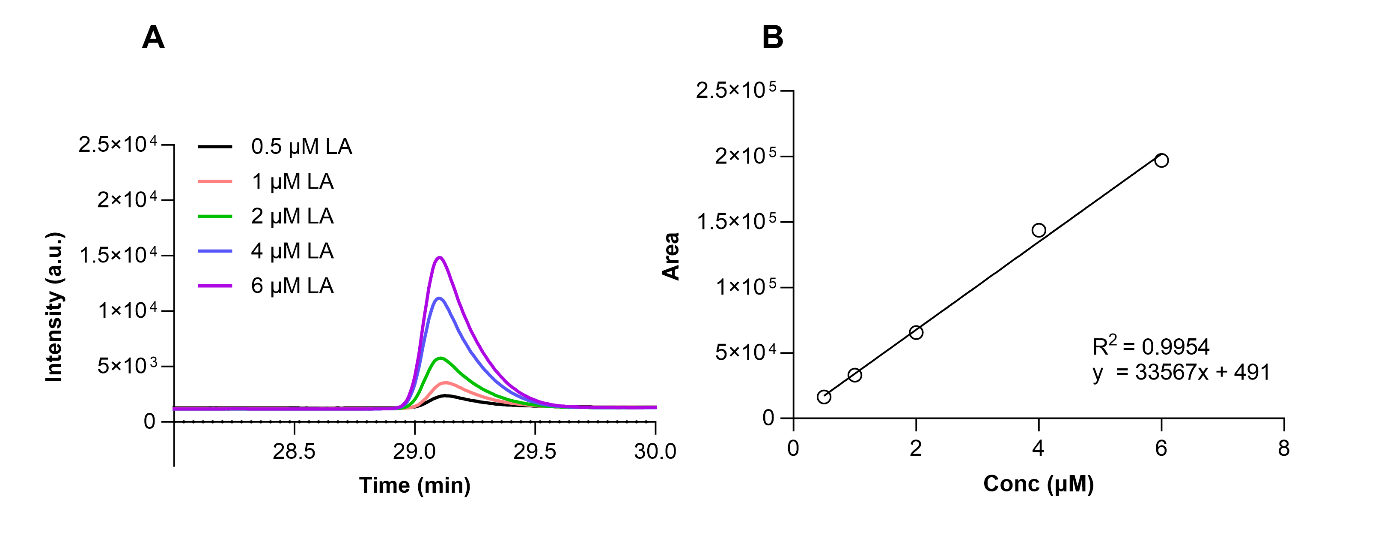


**Supplementary Figure 1: ABD-F-LA calibration curve.** Chromatographic conditions: Kinetex 2.6u C18 100A column, 30 min linear gradient 0 - 50% B using H_2_O-CH_3_CN (5:95) and H_2_O-CH_3_CN (95:5) solutions containing 0,1% trifluoroacetic acid (TFA). Flow rate 0.5 ml/min. Fluorescence intensity was measured with excitation at 380 nm and emission at 510 nm. Calibration curves were made with LabSolutions software 5.42 version (Shimadzu) and visualised in GraphPad Prism 10.

**
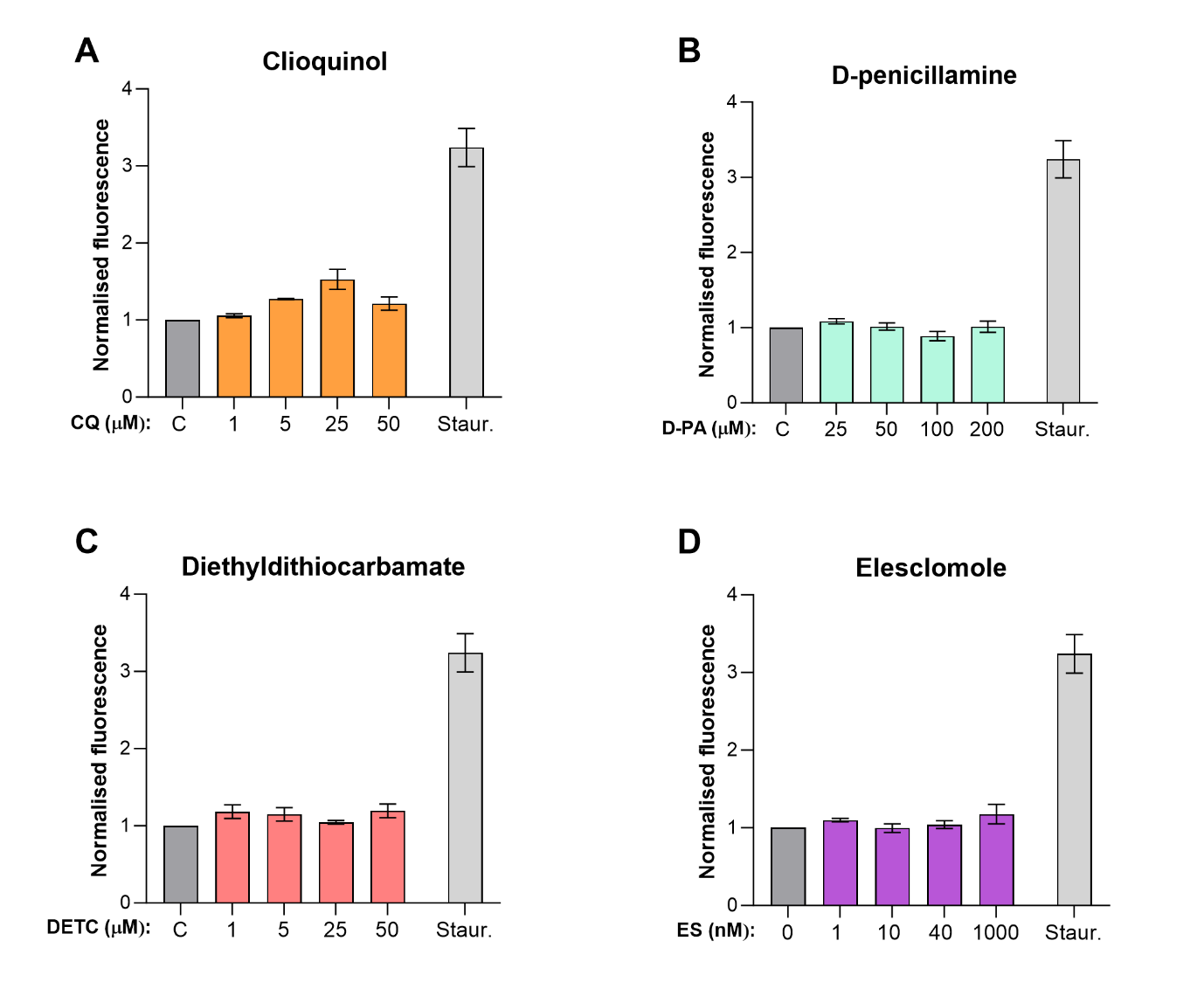
**

**Supplementary figure 2: Toxicity of DETC, CQ, D-PA and ES to differentiated SH-SY5Y cells.** Cells were incubated for 24h with different concentrations (1nM - 200μM) of CQ (A), D-PA(B), DETC (C) and ES (D). PI fluorescence were measured in 612 nm (excitation 540nm). Staurosporine (staur) was used as a positive control of apoptosis.


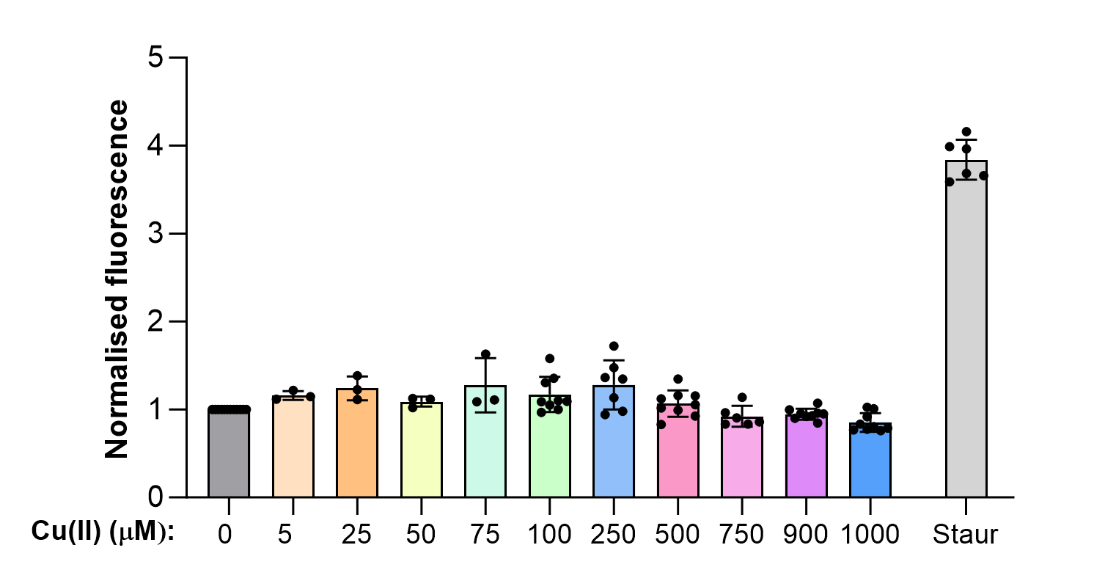


**Supplementary figure 3: Copper toxicity to differentiated SH-SY5Y cells.** Cells were incubated for 24 h with different CuCl_2_ concentrations (5 – 1000 μM). PI fluorescence were measured in 612 nm (excitation 540 nm). Staurosporine (staur) was used as a positive control of apoptosis.


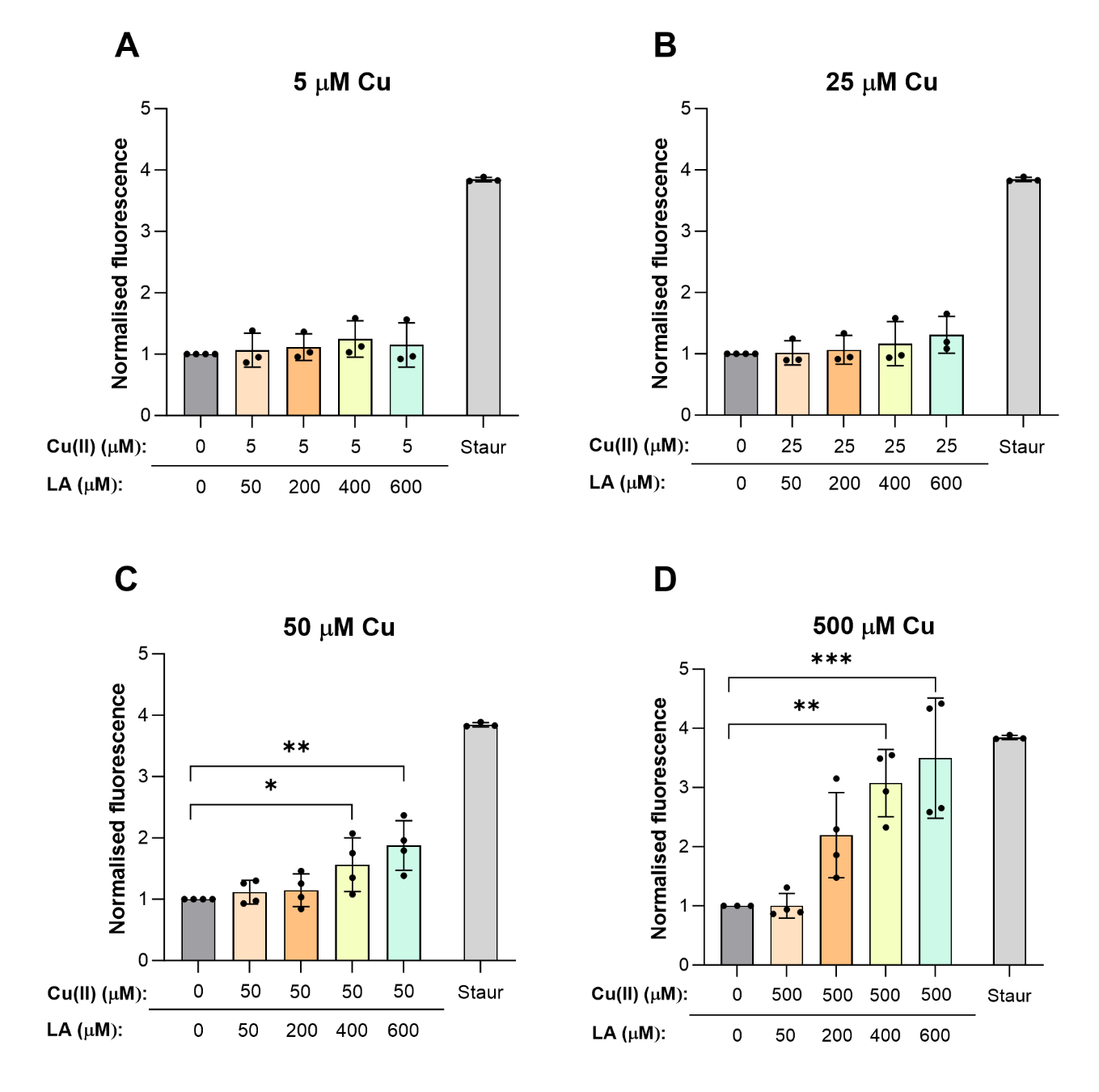


**Supplementary figure 4: Copper toxicity to differentiated SH-SY5Y cells in the presence of LA.** Cells were incubated for 24 h with different LA concentrations (50 – 600 μM) in the presence of 5 μM CuCl_2_ (A); 25 μM CuCl_2_ (B); 50 μM CuCl_2_ (C) and 500 μM CuCl_2_ (D). PI fluorescence wase measured at 612 nm (excitation 540 nm). Staurosporine (staur) was used as a positive control of apoptosis. Data are shown as mean ± SEM; n = 3-4. One-way ANOVA followed by a Dunnett’s multiple comparisons test at the 0.05 level was used for statistical analysis. Main effect of treatment ∗∗∗∗ p < 0.0001; ∗ p < 0.05; n.s., not significant with respect to the cells treated with 5 μM CuCl_2_.

**
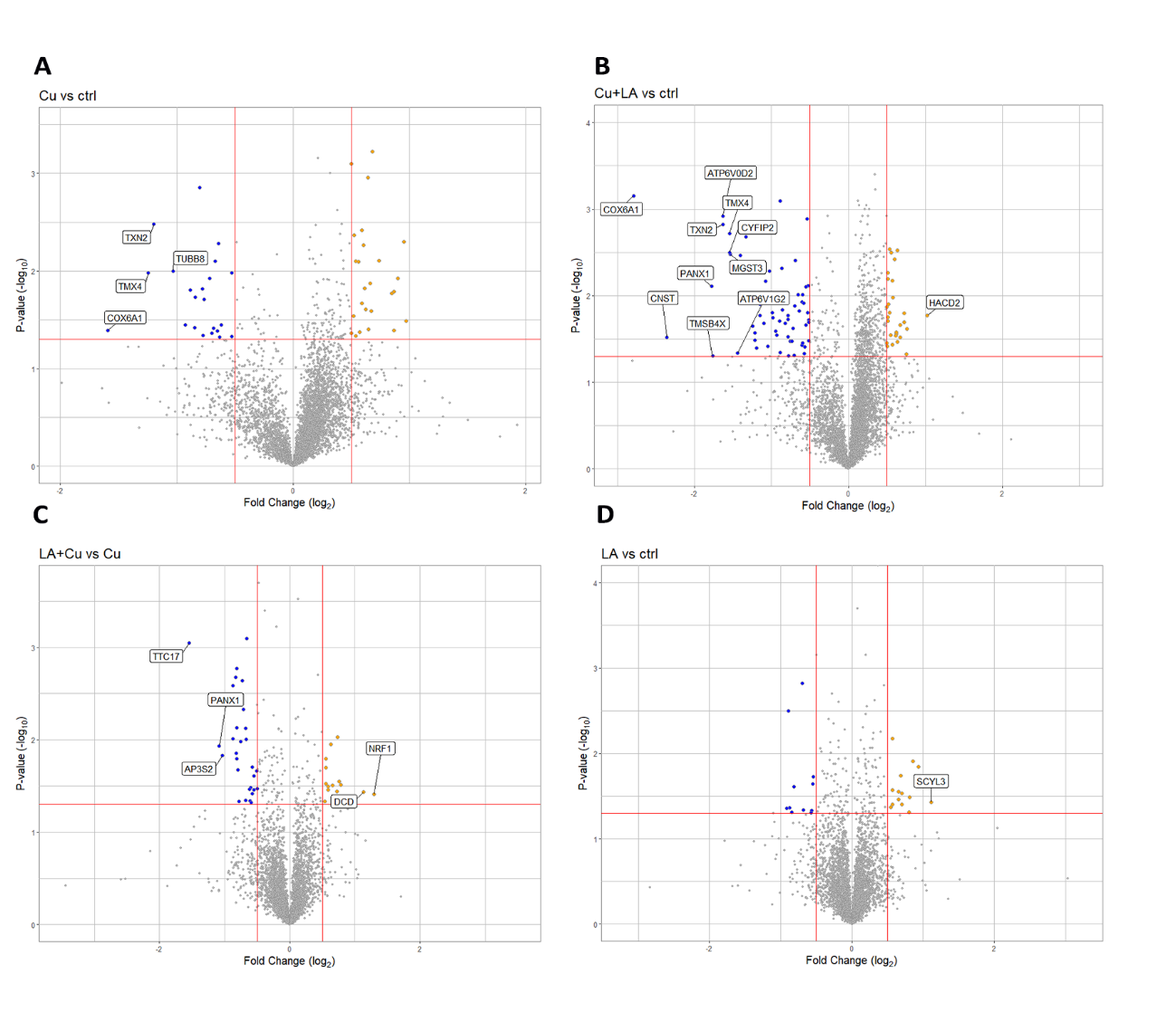
**

**Supplementary Figure 5.** Volcano plots comparing proteome of (A) cells treated with 5 μM CuCl_2_ to non-treated cells, (B) cells treated with 5 μM CuCl_2_ and 20 μM LA to non-treated cells, (C) cells treated with 5 μM CuCl_2_ and 20 μM LA to 5 μM CuCl_2_ -treated cells, and (D) cells treated with 20 μM LA to non-treated cells. Proteins labelled: top 10 up- and down regulated proteins with fold change over 2x (log_2_ < -1 or log_2_ > 1). Plots were generated with R software (ggplot package), cut-off: log_2_ > 0.5, p-value < 0.05 (represented with red lines).

**Supplementary Table 1.** List of proteins involved in cellular Cu-handling machinery detected by LC‑MS/MS analysis of differentiated SH-SY5Y cells with changes after treatment with 5 μM CuCl_2_ and/or 20 μM LA. Values denoted with asterisk (*) were detected only in one biological parallel and cannot be interpreted correctly. Proteins up- or downregulated at least in one treatment group are denoted in bold.

| Protein | Average abundance ratio by comparison group | | | |
| --- | --- | --- | --- | --- |
|  | Cu/control | LA/control | Cu+LA/control | Cu+LA/Cu |
| **ATOX1** | **0.55** | 0.64 | 0.89 | **1.63** |
| ATP7A | 0.96 | 0.98 | 0.98 | 1.02 |
| CCS | 1.03 | 1.08 | 1.02 | 0.99 |
| COX17 | 1.16 | 0.96 | 1.15 | 0.99 |
| SCO1 | 0.94 | 1.07 | 1.02 | 0.93 |
| SCL31A1 (CTR1) | 0.95* | 1.10 | 0.88* | 1.09* |
| SOD1 | 1.04 | 0.99 | 1.14 | 1.10 |

**Supplementary Table 2.** List of top up- and downregulated proteins and expression changes candidates in SH-SY5Y cells treated with 5 µM CuCl_2_ or 20 µM LA, alone or in combination, for 24 h.

| Gene ID | Description | Abundance ratio |
| --- | --- | --- |
| Upregulated proteins in 5 µM CuCl_2_-treated cells compared to n.t. cells (top 10) | | |
| SF1 | Splicing factor 1 | 1.96 |
| DPCD | Protein DPCD | 1.82 |
| CSRP2 | Cysteine and glycine-rich protein 2 | 1.80 |
| L1CAM | Neural cell adhesion molecule L1, isoform 3 | 1.60 |
| PEX1 | Peroxin-1 | 1.57 |
| PLCB3 | 1-phosphatidylinositol 4,5-bisphosphate phosphodiesterase beta-3, isoform 2 | 1.56 |
| RABGGTA | Geranylgeranyl transferase type-2 subunit alpha | 1.54 |
| SH3GLB1 | Endophilin-B1 | 1.53 |
| TBC1D10B | TBC1 domain family member 10B | 1.52 |
| EXOC1 | Exocyst complex component 1, isoform 2 | 1.50 |
| Downregulated proteins in 5 µM CuCl_2_-treated cells compared to n.t. cells (top 10) | | |
| COX6A1 | Cytochrome c oxidase subunit 6A1, mitochondrial | -3.02 |
| TMX4 | Thioredoxin-related transmembrane protein 4 | -2.37 |
| TXN2 | Thioredoxin, mitochondrial | -2.29 |
| TUBB8 | Tubulin beta-8 chain | -2.04 |
| MAP2K7 | Dual specificity mitogen-activated protein kinase kinase 7 | -1.90 |
| TMEM256-PLSCR3 | TMEM256-PLSCR3 readthrough (NMD candidate) | -1.84 |
| MEMO1 | Protein MEMO1 | -1.80 |
| WDR59 | GATOR complex protein WDR59 | -1.79 |
| MTARC1 | Mitochondrial amidoxime-reducing component 1 | -1.74 |
| BRAP | BRCA1-associated protein | -1.72 |
| Upregulated proteins in 5 µM CuCl_2_ and 20 µM LA-treated cells compared to n.t. cells | | |
| CPSF4 | Cleavage and polyadenylation specificity factor subunit 4, isoform 3 | 2.48 |
| HACD2 | Very-long-chain (3R)-3-hydroxyacyl-CoA dehydratase 2 | 2.03 |
| SPOUT1 | Putative methyltransferase C9orf114 | 1.70 |
| SF1 | Splicing factor 1 | 1.69 |
| COMMD7 | COMM domain-containing protein 7 | 1.59 |
| POLR2I | DNA-directed RNA polymerase II subunit RPB9 | 1.56 |
| TIMM10 | Mitochondrial import inner membrane translocase  subunit Tim10 | 1.55 |
| DCTN1 | Dynactin subunit 1 | 1.54 |
| RAB23 | Ras-related protein Rab-23 | 1.54 |
| UBE2R2 | Ubiquitin-conjugating enzyme E2 R2 | 1.53 |
| Downregulated proteins in 5 µM CuCl_2_ and 20 µM LA-treated cells compared to n.t. cells | | |
| COX6A1 | Cytochrome c oxidase subunit 6A1, mitochondrial | -6.93 |
| CNST | Consortin, isoform 2 | -5.14 |
| PANX1 | Pannexin-1, isoform 2 | -3.43 |
| TXN2 | Thioredoxin, mitochondrial | -3.10 |
| ATP6V0D2 | V-type proton ATPase subunit d 2 | -3.10 |
| TMX4 | Thioredoxin-related transmembrane protein 4 | -2.92 |
| CYFIP2 | Cytoplasmic FMR1-interacting protein | -2.92 |
| MGST3 | Microsomal glutathione S-transferase 3 | -2.90 |
| ATP6V1G2 | V-type proton ATPase subunit G 2 | -2.71 |
| TUBB8 | Tubulin beta-8 chain | -2.64 |
| Upregulated proteins in 5 µM CuCl_2_ and 20 µM LA-treated cells compared to 5 µM CuCl_2_-treated cells (top 10) | | |
| NRF1 | Nuclear respiratory factor 1, short isoform | 3.69 |
| DCD | Dermcidin | 2.20 |
| FGF2 | Fibroblast growth factor | 1.69 |
| TELO2 | Telomere length regulation protein TEL2 homolog | 1.66 |
| DNAJC17 | DnaJ homolog subfamily C member 17 | 1.65 |
| SURF6 | Surfeit locus protein 6 | 1.57 |
| CRMP1 | Dihydropyrimidinase-related protein 1 | 1.55 |
| FIBP | Isoform Short of Acidic fibroblast growth factor intracellular-binding protein | 1.51 |
| SAP30 | Histone deacetylase complex subunit SAP30 | 1.50 |
| TBCC | Tubulin-specific chaperone C | 1.47 |
| Downregulated proteins in 5 µM CuCl_2_ and 20 µM LA-treated cells compared to 5 µM CuCl_2_-treated cells (top 10) | | |
| FAM114A2 | Protein FAM114A2 | -1.83 |
| RABGGTA | Geranylgeranyl transferase type-2 subunit alpha | -1.83 |
| CHMP7 | Charged multivesicular body protein 7 | -1.76 |
| HNRNPK | Heterogeneous nuclear ribonucleoprotein K | -1.74 |
| KMT2A | Histone-lysine N-methyltransferase 2A, isoform 2 | -1.72 |
| PRRC1 | Protein PRRC1 | -1.69 |
| RNF13 | E3 ubiquitin-protein ligase RNF13 | -1.63 |
| PSMG1 | Proteasome assembly chaperone 1 | -1.60 |
| IDH3G | Isocitrate dehydrogenase [NAD] subunit gamma, mitochondrial | -1.59 |
| TTC9C | Tetratricopeptide repeat protein 9C | -1.58 |
| Upregulated proteins in 20 µM LA-treated cells compared to n.t. cells (top 10) | | |
| SCYL3 | Protein-associating with the carboxyl-terminal domain of ezrin | 2.17 |
| BPI | Bactericidal permeability-increasing protein | 2.11 |
| ARFIP1 | Arfaptin-1, isoform A | 1.91 |
| SAMD4B | Protein SMAUG homolog 2 |  |
| PARG | Poly(ADP-ribose) glycohydrolase | 1.75 |
| MYL6B | Myosin light chain 6B | 1.63 |
| IGBP1 | Immunoglobulin-binding protein 1 | 1.63 |
| RLG2 | Ral guanine nucleotide dissocication stimulator-like 2 | 1.61 |
| TBCEL | Tubulin-specific chaperon cofactor E-like protein | 1.61 |
| QRSL1 | Glutamyl-tRNA (Gln) amidotransferase subunit A, mitochondrial | 1.60 |
| Downregulated proteins in 20 µM LA-treated cells compared to n.t. cells (top 10) | | |
| MAP2K7 | Dual specificity mitogen-activated protein kinase kinase 7 | -1.89 |
| C1orf131 | Uncharacterized protein C1orf131 | -1.86 |
| BLOC1S2 | Biogenesis of lyzosome-related organelles complex 1 subunit 2 | -1.84 |
| MPV17 | MpV17 transgene, murine homolog, glomerulosclerosis, isoform CRA_f | -1.79 |
| CNNM3 | Metal transporter CNNM3, isoform 2 | -1.76 |
| ABR | Active breakpoint cluster region-related protein | -1.62 |
| MTCH1 | Mitochondrial carrier homolog 1 | -1.61 |
| USP4 | Ubiquitin carboxyl-terminal hydrolase 4, isoform 2 | -1.49 |
| ARHGEF2 | Guanine nucleotide exchange factor H1 | -1.48 |
| NME6 | Nucleoside diphosphate kinase | -1.46 |
